# Dual-Slope Path Loss Model for Integrating Vehicular Sensing Applications in Urban and Suburban Environments

**DOI:** 10.3390/s24134334

**Published:** 2024-07-04

**Authors:** Herman Fernández, Lorenzo Rubio, Vicent M. Rodrigo Peñarrocha, Juan Reig

**Affiliations:** 1 Telecommunications Research Group, Pedagogical and Technological University of Colombia, Sogamoso 152211, Colombia; 2 Antennas and Propagation Lab, Universitat Politècnica de València, 46022 Valencia, Spain; lrubio@dcom.upv.es (L.R.); vrodrigo@dcom.upv.es (V.M.R.P.); jreigp@dcom.upv.es (J.R.)

**Keywords:** vehicular ad hoc network (VANET), vehicle-to-everything (V2X), artificial intelligence (AI), internet of things (IoT), path loss models, path loss exponent, 5G, autonomous driving (AD), cooperative autonomous driving (CAD), cooperative sensing, connected and autonomous vehicles (CAVs)

## Abstract

The development of intelligent transportation systems (ITS), vehicular ad hoc networks (VANETs), and autonomous driving (AD) has progressed rapidly in recent years, driven by artificial intelligence (AI), the internet of things (IoT), and their integration with dedicated short-range communications (DSRC) systems and fifth-generation (5G) networks. This has led to improved mobility conditions in different road propagation environments: urban, suburban, rural, and highway. The use of these communication technologies has enabled drivers and pedestrians to be more aware of the need to improve their behavior and decision making in adverse traffic conditions by sharing information from cameras, radars, and sensors widely deployed in vehicles and road infrastructure. However, wireless data transmission in VANETs is affected by the specific conditions of the propagation environment, weather, terrain, traffic density, and frequency bands used. In this paper, we characterize the path loss based on the extensive measurement campaign carrier out in vehicular environments at 700 MHz and 5.9 GHz under realistic road traffic conditions. From a linear dual-slope path loss propagation model, the results of the path loss exponents and the standard deviations of the shadowing are reported. This study focused on three different environments, i.e., urban with high traffic density (U-HD), urban with moderate/low traffic density (U-LD), and suburban (SU). The results presented here can be easily incorporated into VANET simulators to develop, evaluate, and validate new protocols and system architecture configurations under more realistic propagation conditions.

## 1. Introduction

The development of intelligent transportation systems (ITS), vehicular ad hoc networks (VANETs), and autonomous driving (AD) has accelerated in recent years, driven by artificial intelligence (AI), the internet of things (IoT), and their integration with dedicated short-range communications (DSRC) systems and fifth-generation (5G) networks since 5G is emerging as a platform for connecting sensors and vehicles on the road, providing vehicle-to-everything (V2X) services to drivers and pedestrians [1,2,3,4]. This is due to the potential of IoT-focused applications of AD and the use of 5G New Radio interfaces to meet these integration requirements given the use scenarios defined in the ITU-M2150-1 recommendation for IMT-2020 systems [5]. These include enhanced mobile broadband (eMBB), ultra-reliable low-latency communication (URLLC), and massive machine-type communication (mMTC). In [6], the authors also described how services such as autonomous vehicles (AV), ITS, V2X, industry 4.0, and smart grid are related to URLLC. This leads to improved mobility conditions in the different road propagation environments, including urban, suburban, rural, and highway, among others. The integration of ITS, VANETs, AD, AV, and V2X with the URLLC scenario has rendered drivers and pedestrians more aware of the need to improve their behavior and decision making in adverse traffic conditions by enabling them to exchange information from different types of sensors widely deployed in vehicles and road infrastructure. However, the wireless data transmission in these systems is affected by the specific conditions of the propagation environment, including the weather, orography, traffic density, and frequency bands used.

The future design of vehicle communication systems has received considerable attention in recent years from the automotive industry, government, and the scientific community as the integration of vehicle-to-vehicle (V2V) and vehicle-to-infrastructure (V2I). These are aimed at proposing better vehicle-reliable safety applications based on DSRC [2], which require high reliability in the connectivity between V2V and V2I communications. In this sense, various types of sensors such as cameras, radars, and speed detectors, among others, have been implemented to support the development of AD and improve mobility conditions in cities and on the road network [4,7,8]. For example, in [4], an integrated sensing and communication system (ISAC) was proposed based on a path loss prediction approach to improve the knowledge of wireless data transmission in vehicular networks. The approach predicts the end-to-end path loss distribution using multimodal data collected by millimeter wave (mmWave) radars, laser radars, and cameras. The important role of sensor fusion in intelligent transportation systems was described in [7]. This paper provides a comprehensive overview of the capabilities, the impacts, the planning, and the technological challenges of AVs. In [8], the author introduced several V2X use cases for autonomous driving, where cooperative autonomous driving is categorized into two types: cooperative sensing and cooperative decision. Cooperative sensing focuses on the exchange of sensor information between V2V and V2I. This will further enhance road safety, reduce traffic congestion, and improve travel comfort in urban environments [9,10]. In this sense, it is necessary to develop measurement campaigns in vehicular propagation channels in order to provide more accurate propagation models that can be used to determine the parameters in the characterization of the propagation channel.

Cooperative sensing uses VANET simulators to develop, evaluate, and validate new protocols and system architecture configurations under more realistic propagation conditions. Therefore, it is necessary to propose dual-slope models that allow for more efficient radio planning. Dual-slope models consider a breaking point or critical distance that depends on factors such as the propagation scenario and the frequency band used, among others. This critical distance indicates the end of one wave propagation mechanism and the beginning of another, where more path loss can appear. Dual-slope propagation models allow the generation of results that can be easily incorporated into VANET simulators to optimize the deployment of sensors in the road infrastructure networks.

Government standards bodies have identified specific bands for the development of ITS applications. For example, the Federal Communication Commission (FCC) issued a report and order adopting rules that repurposed the 5850–5895 MHz to expand unlicensed mid-band operations while continuing to dedicate the 5895–5925 MHz for ITS operations [11]. Also, the FCC has proposed that the transition of ITS operations from DSRC-based technology to cellular V2X (C-V2X)-based technology will occur in accordance with a schedule to be determined in a future report and order. In Europe, the European Telecommunication Standard Institute (ETSI) has adopted the DSRC band for ITS applications, allocating 50 MHz (5.875 to 5.925 GHz) [12]. The specific characteristics of both applications (safety and non-safety) require the development and implementation of new communication technologies, where the characterization and modeling of the vehicle channel play a very important role [13,14]. Although progress has been made in the characterization of the V2X radio channel in recent years [15,16], more research is needed that focuses on future vehicle networks that enable interaction with the 5G IoT ecosystem.

Several path loss models have been developed for vehicular network scenarios. These include the one/dual-slope, ray tracing (RT), floating intercept (FI), close-in (CI), free space reference distance, and ABG models. For example, large-scale path loss models for urban environments have been proposed based on the CI, FI, and ABG models under line-of-sight (LOS) and non-line-of-sight (NLOS) conditions in mmWave frequency bands [17]. In [18], the authors presented a shadow fading model for system simulations based on real measurements in urban and highway scenarios. The measurement data were divided into three categories, namely LOS, obstructed line-of-sight (OLOS) by vehicles, and NLOS, and it was observed that vehicles obstructing the LOS induce an additional average attenuation of about 10 dB in the received signal power. In addition, a connection probability in V2V urban scenarios was analyzed based on a dual-slope path loss model on both LOS and OLOS [19]. In [20], path loss models were proposed considering weather conditions, and RT simulations were performed to verify the accuracy of the proposed expression. In [21], the authors adopted a measurement-based dual-slope path loss model to analyze the channel capacity performance for both direct transmission of inter-vehicle and infrastructure-based cooperative communication in VANETs. On the other hand, Wei and Tao analyzed the effects of antenna height on V2I communication in rural areas. They first classified the V2I communication into LOS and NLOS links and then established two-beam ground reflection and integrated models for LOS and NLOS conditions, respectively [22]. In [23], a two-slope model was proposed by performing path loss measurements in a sports utility vehicle (SUV) at 915 MHz and 2.4 GHz. On the other hand, ref. [24] provided an overview of experimentally verified propagation models for wireless sensor networks (WSNs) and quantitative comparisons of propagation models used in WSN research under different scenarios and frequency bands.

Furthermore, the large differences between V2V propagation channels and fixed-to-mobile (F2M) propagation channels, transmitter (Tx) and receiver (Rx) heights, propagation environments, and frequency bands mean that the propagation models developed for the deployment of F2M systems cannot be applied in the performance evaluation and development of future ITS applications over VANETs, thus forcing characterization and modeling of the radio channel at 700 MHz and in the DSRC band at 5.9 GHz for future V2X systems.

This paper presents the vehicular channel characterization by using the dual-slope path loss propagation model in urban with high traffic density (U-HD), urban with low traffic density (U-LD), and suburban (SU) scenarios in the city of Valencia, based on real propagation measurements carried out in 2012 at 700 MHz and 5.9 GHz. The paper is organized as follows: Section 2 describes the measurement system and the main characteristics of the propagation scenarios where the measurements were performed. Section 3 presents the results derived from the analysis of the measurements, where the path loss exponent (PLE) behavior of the received signal is analyzed and classified according to the propagation scenarios. Finally, the conclusions derived from the study are summarized in Section 4.

## 2. Methodology

### 2.1. Measurement System

**Figure 1:**
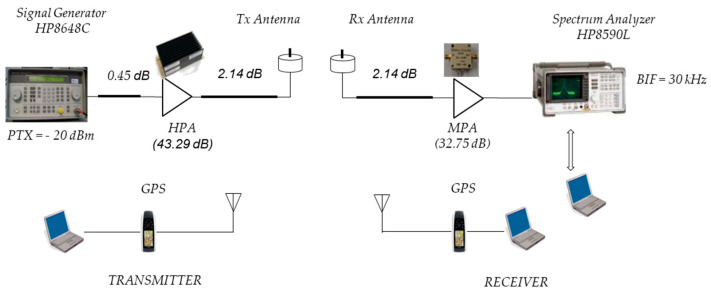
Channel sounder at 700 MHz.

The channel sounder implemented at 700 MHz is shown in Figure 1. This channel sounder consists of an Hewlett HP8648C signal generator in the Tx vehicle transmitting an unmodulated carrier at 700 MHz and a Hewlett Packard HP8590L spectrum analyzer (SA) in the Rx vehicle. A SPAN of zero was selected in the SA to measure received power on 401-point traces. The antennas used are monopoles with a horizontal plane gain of approximately −5.43 dB. For transmission, a power amplifier was used that allowed transmission with an equivalent isotropic radiated power (EIRP) of 26.3 dBm. For reception, an amplifier with a gain of 32.75 dB was used.

**Figure 2:**
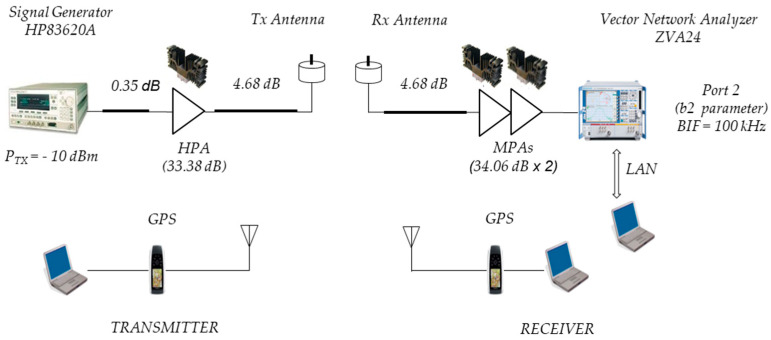
Channel sounder at 5.9 GHz.

The channel sounder implemented at 5.9 GHz is shown in Figure 2. This channel sounder consists of a Hewlett Packard HP83623A signal generator in the Tx vehicle, which transmits an unmodulated carrier at 5.9 GHz, and the Rohde & Schwartz ZVA24 vector network analyzer (VNA) in the Rx vehicle. The VNA was used in power meter mode, directly measuring the b_2_ parameter in traces of 5000 points. The antennas used in Tx/Rx are monopoles at λ/4 with a gain in the horizontal plane of about −2.56 dB and a scattering parameter $S_{11}$ lower than −22 dB. A power amplifier was used for transmission, allowing transmission with an EIRP of 23.8 dBm. For reception, two amplifiers were used in series with a total gain of 68.12 dB.

The vehicles were equipped with global position systems (GPS) receivers controlled by laptops to obtain information on the time of measurement, relative speed, and Tx-Rx separation distance. All laptops were synchronized in time to correlate the measurements taken by the VNA with the information provided by the GPS receivers.

**Figure 3:**
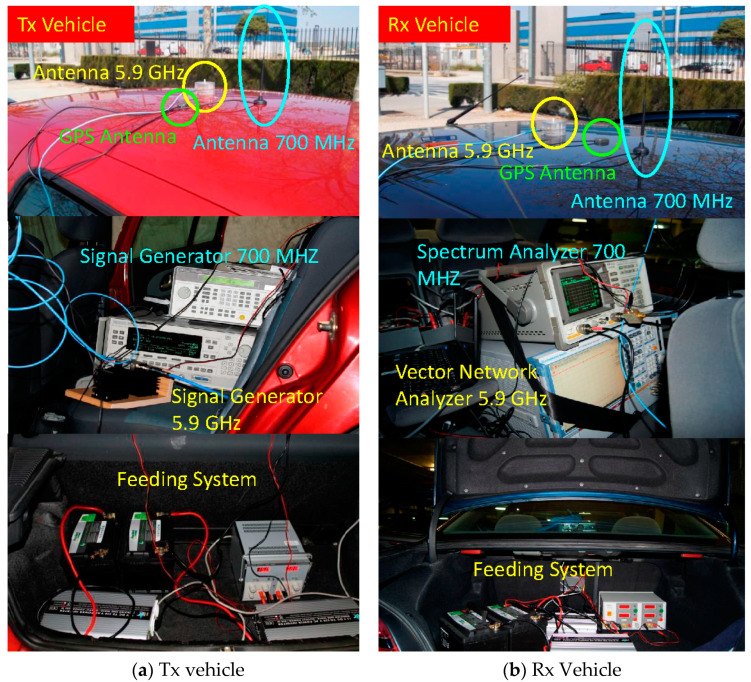
Tx/Rx vehicles together with on-board equipment at 700 MHz and 5.9 GHz.

The vehicles used for the measurements were a Renault (Tx) (Figure 3a) and a Peugeot (Rx) (Figure 3b). The antennas were mounted on the roof of the vehicles at a height of approximately 1.41 m and 1.45 m above the ground for Tx and Rx, respectively. Part of the on-board equipment is also shown in Figure 3. In each of the vehicles, 75 Ah batteries and 12 V DC to 220 V AC inverters were used as the power supply system, allowing an autonomous time of approximately 90 min.

### 2.2. Scenarios

**Figure 4:**
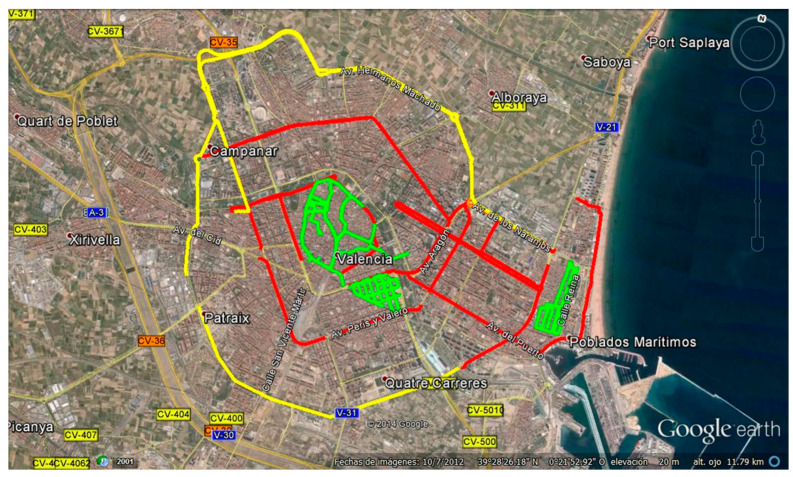
Scenario 1 is shown with a red line, scenario 2 is shown with a green line, and scenario 3 is shown with a yellow line.

The type of environment, vehicle speed, and road traffic density determine the propagation characteristics of the vehicle channel. Traffic density and vehicle speed are usually higher in suburban environments. These conditions allowed the selection of the most suitable areas for the measurement campaign to characterize the path loss propagation. A series of avenues and ring roads were found, forming a concentric ring around the city of Valencia, which, according to its characteristics, was defined as a suburban environment and is referred to as scenario 3. Figure 4 illustrates the routes taken in this scenario with a yellow line. Moving inland in the concentric rings, a clearly urban environment with a high road traffic density is presented and referred to as scenario 1. The routes taken are shown with a red line in Figure 4. Within this urban environment, some areas with particular and interesting characteristics were selected. Thus, we analyzed the area of the old town of the city, where narrow streets without a defined shape meet small squares, abrupt intersections, and cobbled pedestrian streets with a large absence of pedestrians. We also utilized a special area within this old town, such as the Plaza del Ayuntamiento. Figure 4 shows in green the routes taken in these urban environments with low road traffic density, which are referred to as scenario 2.

**Figure 5:**
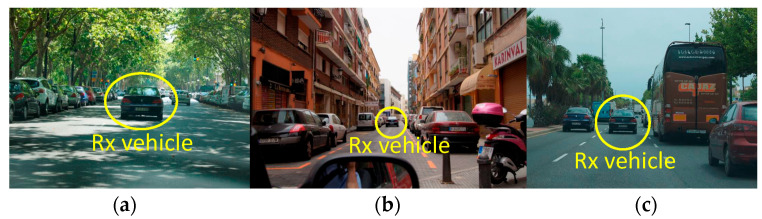
Measurement scenarios, (**a**) urban high density, (**b**) urban low density, and (**c**) suburban. The Rx vehicle can be seen driving in front of the Tx vehicle.

Scenario 1: This is an urban environment with high traffic density, with an average of 44,200 vehicles/24 h. Two avenues in the city of Valencia were chosen for the analysis, namely Avenida del Puerto (one-way traffic) and Avenida Blasco Ibañez (two-way traffic), with four and five lanes in each direction, respectively. Figure 5a shows a view of the measurement scenario.

Scenario 2: This is the urban center of the city of Valencia, the old town, with low traffic density at an average of 7500 vehicles/24 h and with narrow streets and one-way traffic. The streets are approximately 10 meters wide, including, in some cases, parking lots and sidewalks on both sides of the street. Figure 5b shows a view of the measurement scenario.

Scenario 3: This scenario is the Ronda Norte in the city of Valencia, Spain, with an average intensity of 71,200 vehicles/24 h, with three and four lanes of traffic in each direction, with wide open spaces on both sides alternating with buildings close to the roadway and medium-height trees along several sections of the avenue. Figure 5c shows a view of the measurement scenario.

It is relevant to note that the measurements were carried out at 700 MHz and 5.9 GHz (DSRC band), with the Tx and Rx vehicles traveling in the same direction several times over the measurement scenario on weekdays between 10:00 and 13:00. Measurements were also carried out under normal driving conditions, with alternations of LOS and NLOS between the Tx vehicle and the Rx vehicle.

## 3. Link Budget and Dual-Slope Path Loss Measurement Results

The path loss is one of the most significant parameters in radio link design and provides a measure of channel quality. It is expressed as the average level of path loss in dB and varies as a function of the Tx and Rx separation distance. The path loss considers the propagation mechanisms present in the radio channel, such as free space, rejection, diffraction and scattering, the influence of the propagation environments (urban, suburban, rural, and highway), directional characteristics, antenna heights, and Tx and Rx separation distance. In addition, the signal-to-noise ratio (SNR) is inversely related to the path loss; i.e., as the path loss increases, the SNR at the Rx decreases, and the coverage area is reduced.

The average received power in log units (dBm) under free space propagation conditions, denoted by $\;P_{Rx}\left(d\right)$, is given by the following:

(1) $$P_{Rx}\left(d\right)=P_{Tx}+G_{Tx}+G_{Rx}-10log_{10}{\left(\frac{4\pi d}{\lambda_{c}}\right)}^{2},$$

where $P_{Tx}$ is the transmitted power in dBm, d is the Tx/Rx separation distance; $G_{Tx}$ and $G_{Rx}$ are the transmit and receive antennas’ gain expressed in decibels (dB), respectively; and $\lambda_{c}$ is the wavelength associated with the carrier frequency $f_{c}$. The last term in Equation (1) is the path loss for free space propagation conditions, $PL_{FS}\left(d\right)$, expressed in dB.

### 3.1. Link Budget at 5.9 GHz and at 700 MHz

**Figure 6:**
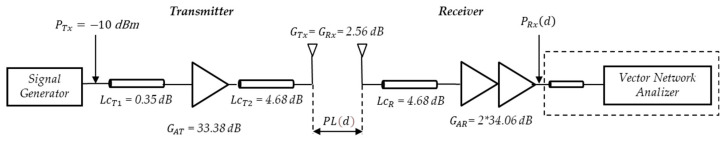
V2V schematic diagram at 5.9 GHz.

Figure 6 shows the schematic diagram of the 5.9 GHz V2V measurement system and the components used to perform the link budget.

According to (1) and taking the received power level $P_{Rx}\left(d\right)$ as that related to the VNA input with $P_{Tx}$= −10 dBm and subtracting the cable losses $Lc_{T1}$ = 0.35 dB, $Lc_{T2}$ = 4.68 dB, and $Lc_{R}$ = 4.68 dB and then adding the gains of the amplifier $G_{AT}$ = 33.38 dB in Tx and the two cascaded amplifiers $G_{AR}$ = 2 × 34.06 dB in Rx and the Tx and Rx antenna gains $G_{Tx}$ = $G_{Rx}$ = 2.56 dB, the value of $P_{Rx}\left(d\right)$ is given by the following:

(2) $$P_{Rx}\left(d\right)=P_{Tx}-Lc_{T1}+G_{AT}-Lc_{T2}+G_{Tx}+G_{Rx}-PL\left(d\right)-Lc_{R}+G_{AR}.$$

Therefore, the path loss propagation at 5.9 GHz, expressed in (dB) and denoted by $PL\left(d\right)$, is given by the following:

(3) $$PL\left(d\right)=76.67-P_{Rx}\left(d\right).$$

Using the parameter $b_{2}\left(f\right)$ measured by the VNA as the received power value, records were obtained to analyze the behavior of the path loss as a function of d.

**Figure 7:**
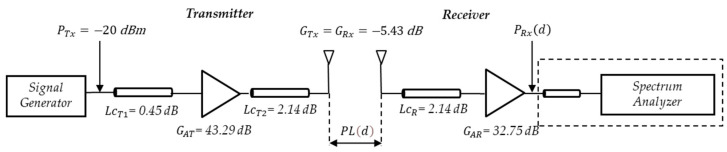
V2V schematic diagram at 700 MHz.

The link budget for the 700 MHz test system was performed in the same manner as for the 5.9 GHz test system, as shown in Figure 7.

Taking the received power level $P_{Rx}\left(d\right)$ with respect to the SA input and also, taking into account a $P_{Tx}$ = −20 dBm and subtracting the cable losses $Lc_{T1}$ = 0.45 dB, $Lc_{T2}$ = 2.14 dB, and $Lc_{R}$ = 2.14 dB and then adding the gains of the amplifier $G_{AT}$ = 43.29 dB in Tx and the amplifier $G_{AR}$ = 32.75 dB in Rx and the Tx and Rx antenna gains $G_{Tx}$ = $G_{Rx}$ = −5.43 dB gives the same expression as in Equation (2). In this case, the $PL\left(d\right)$ at 700 MHz is expressed (in dB) by the following:

(4) $$PL\left(d\right)=40.45-P_{Rx}\left(d\right).$$

Table 1 summarizes the main parameters used to configure the measurement systems.

### 3.2. Dual-Slope Path Loss Model Results

To process the data sets obtained during the measurement campaign, a selection was made according to the propagation scenarios, analyzing the behavior of the path loss propagation as a function of the separation distance between the Tx and Rx. A further process of data filtering was carried out to obtain more accurate values for the parameters of the dual-slope path loss model. According to [25], a linear relationship can be established between the mean path loss expressed in logarithmic units, $PL\left(d\right)$, and $log_{10}\left(d\right)\;$ in V2V radio channels, analogous to traditional F2M channels, given by the following:

(5) $$\begin{array}{cc} PL\left(d\right)=L_{0}+10\gamma log_{10}\left(\frac{d}{d_{0}}\right)+S, & d\geq d_{0}, \end{array}$$

where $L_{0}$ represents the average path loss propagation at a separation distance $d_{0}$, the term $10\gamma log_{10}\left(\frac{d}{d_{0}}\right)$ refers to the average path loss propagation referring to the Tx/Rx distance $d_{0}$, γ is the PLE related to the type of propagation environment, and S is a Gaussian distributed random variable with zero mean and standard deviation $\;\sigma_{S}$ in dB that is used to model long-term fading or shadowing.

However, there are environments in which a dual-slope model can more accurately fit the measured data. A dual-slope model is characterized by a path loss exponent of $\gamma_{1}$ and a standard deviation of $\sigma_{S_{1}}\;$ above a reference distance up to critical distance $d_{C}$, a path loss exponent of $\gamma_{2}$, and a standard deviation of $\sigma_{S_{2}}\;$ for a distance higher than the critical distance. Using this model, the average path loss value can be estimated as the following:

(6) $$PL\left(d\right)\left[dB\right]=\left\{\begin{array}{c} \begin{array}{cc} L_{0}+10\gamma_{1}log_{10}\left(\frac{d}{d_{0}}\right)+S, & d_{0}\leq d\leq d_{C}; \end{array}\\ \begin{array}{cc} L_{0}+10\gamma_{1}log_{10}\left(\frac{d_{C}}{d_{0}}\right)+10\gamma_{2}log_{10}\left(\frac{d}{d_{C}}\right)+S, & d\geq d_{C}. \end{array} \end{array}\right.$$

On the other hand, the behavior of the path loss propagation based on a dual-slope model was observed for some of the analyzed routes. It should be noted that this behavior did not appear in all propagation scenarios or in the two frequency bands (700 MHz and 5.9 GHz) in which the measurements were made.

**Figure 8:**
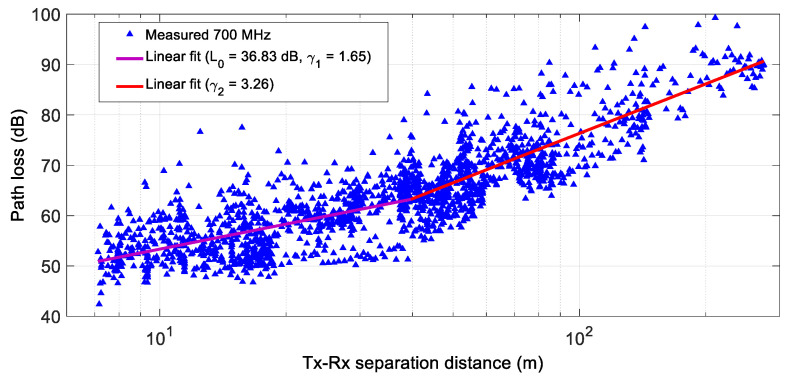
Path loss as a function of Tx/Rx separation distance for an urban environment with high road traffic density at 700 MHz.

Figure 8 shows the path loss as a function of the Tx/Rx separation distance for an urban environment with high traffic density at 700 MHz. A double-slope behavior was observed; thus, applying a linear least squares fit (magenta curve for the first slope and red curve for the second slope in Figure 8) yields values of $L_{0}$ = 36.83 dB, $\gamma_{1}$ = 1.65, $\sigma_{S_{1}}$ = 4.73 dB, $\gamma_{2}$ = 3.26, and $\sigma_{S_{2}}$ = 5.1 dB, with a $d_{C}\;$ = 39.67 m.

**Figure 9:**
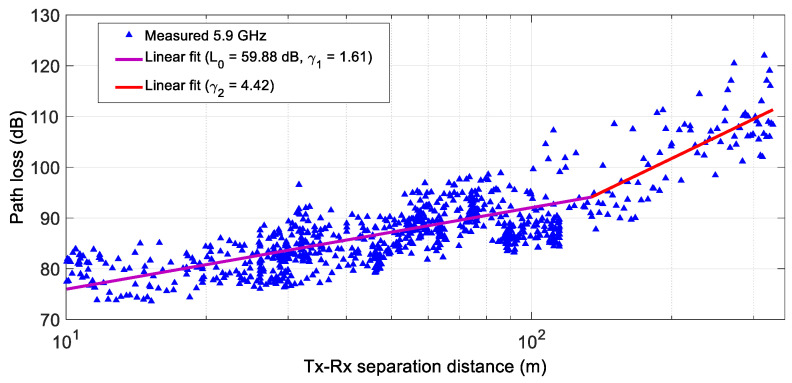
Path loss as a function of Tx/Rx separation distance for an urban environment with low road traffic density at 5.9 GHz.

Similarly, Figure 9 shows the path loss as a function of the Tx/Rx separation distance for an urban environment with low traffic density at 5.9 GHz. We thus obtained $L_{0}$ = 59.88 dB, $\gamma_{1}$ = 1.61, $\sigma_{S_{1}}\;$ = 4.00 dB, $\gamma_{2}\;$ = 4.42, $\sigma_{S_{2}}$ = 5.26 dB, and $d_{C}$ = 134.56 m.

The mean parameters that characterize the path loss propagation for the dual-slope model are summarized in Table 2. The PLE values for the first slope range from 1.04 to 2.13 and 1.72 to 2.46 at 700 MHz and 5.9 GHz, respectively. The highest PLE values for the first slope occurred in urban environments with low traffic density at 700 MHz and 5.9 GHz. Similarly, the PLE values for the second slope range from 3.43 to 4.92 and from 6.26 to 9.69 at 700 MHz and 5.9 GHz, respectively. In addition, the largest exponents of PLE for the second slope occurred in suburban environments at 700 MHz and 5.9 GHz. Note that for the suburban environment at 700 MHz and 5.9 GHz, constructive interference was observed for the first slope due to the multipath effect. This was also observed for the urban environment with high traffic density at 700 MHz.

With respect to the large-scale fading modeled by the parameter $\sigma_{S}$, the received signal suffered the highest variations in urban with low traffic density and suburban environments at 700 MHz and 5.9 GHz. On the other hand, the critical distance ranges from 35.16 m to 58.40 m at 700 MHz, with the largest critical distance obtained in a suburban environment, while the critical distance at 5.9 GHz varies from 132.42 m to 306.04 m for urban with low traffic density and suburban environments, respectively.

It should be noted that in our measurement campaign, there are very few records and scenarios where dual-slope behavior occurred in the path loss propagation analysis. Similarly, only a very small number of researchers have shown results from dual-slope models for vehicular applications [18,26,27,28,29,30]. Table 3 summarizes the values of the dual-slope path loss exponent and the standard deviations of shadowing derived from channel measurement campaigns conducted in different vehicular environments. These results can be used in vehicular network simulators to design, evaluate, and validate new protocols that improve the quality of vehicular communication systems and enable the integration of ITS, AD, and vehicular sensing with the 5G-IoT ecosystem.

The results obtained in this work show the influence of the frequency band and the propagation environment on the estimated values of the path loss propagation and the critical distance. Therefore, when deploying sensor networks for ITS applications in real conditions, the propagation scenario must be considered. According to the results presented in this paper, it is necessary to take into account that urban scenarios with low traffic density have more path loss propagation values for the first slope, up to the critical distance. Similarly, it should be noted that the path loss increased significantly from the critical distance for all propagation environments analyzed in this study at 700 MHz and 5.9 GHz. 

## 4. Conclusions

The results obtained for a dual-slope path loss propagation model in three different environments, i.e., urban with high traffic density, urban with low traffic density, and suburban, are herein presented. These results can be used in vehicular network simulators to design, evaluate, and validate new protocols that improve the quality of vehicular communication systems and enable the integration of ITS, AD, and vehicular sensing with the 5G-IoT ecosystem.

From the results obtained, it should be noted that the parameters of the dual-slope path loss propagation model are influenced by the frequency band. For example, in the results reported in this paper, it was observed that the mean values of the first- and second-slope PLEs were lower at 700 MHz than at 5.9 GHz for urban with low density and suburban environments studied, where the highest values of the first- and second-slope PLEs were obtained for the urban environments with low traffic density at 5.9 GHz.

Future lines of work will include performing broadband measurement campaigns for applications in the ITS to generate path loss and capacity models that enable the optimal deployment of device and sensor networks in V2X 5G-IoT ecosystem.

## Figures and Tables

**Table 1:** Measurement system configuration parameters at 700 MHz and 5.9 GHz.

Parameter	Channel Sounder at 700 MHz	Channel Sounder at 5.9 GHz
Transmitted power	−20 dBm	−10 dBm
Points number	401	5000
Resolution bandwidth	30 kHz	100 kHz
SPAN	0	0
Dynamic range	80 dB	80 dB

**Table 2:** Parameters obtained at 700 MHz and 5.9 GHz.

**Scenario**	**700 MHz**					
	$\gamma_{1}$	$L_{0}$ **(dB)**	$\sigma_{S_{1}}$ **(dB)**	$\gamma_{2}$	$\sigma_{S_{2}}$ **(dB)**	$d_{C}$ **(m)**
1 (Urban H-D)	1.45	37.34	4.52	3.43	5.00	35.16
2 (Urban L-D)	2.13	31.42	5.47	3.76	6.66	35.44
3 (Suburban)	1.04	38.78	4.28	4.92	5.67	58.40
**Scenario**	**5.9 GHz**					
	$\gamma_{1}$	$L_{0}$ **(dB)**	$\sigma_{S_{1}}$ **(dB)**	$\gamma_{2}$	$\sigma_{S_{2}}$ **(dB)**	$d_{C}$ **(m)**
2 (Urban L-D)	2.46	45.88	4.49	6.26	5.01	132.42
3 (Suburban)	1.72	52.40	4.80	9.69	5.25	306.04

**Table 3:** Large-scale dual-slope path loss model parameters for vehicular environments.

Scenario	$\gamma_{1}$	$\sigma_{S_{1}}$ (dB)	$\gamma_{2}$	$\sigma_{S_{2}}$ (dB)	$d_{C}$ (m)	Frequency Band	Related Works
Suburban	2.00–2.10	5.60–2.60	3.80–4.00	4.40–8.40	100	5.9 GHz	Ref. [26]
Highway	1.90	2.50	4.00	0.90	220	5.9 GHz	Ref. [27]
Rural	2.30	3.20	4.00	0.40	226		
Urban intersection 1	1.54	3.64	3.96	4.81	24.5	5.9 GHz	Ref. [28]
Urban intersection 2	1.56	3.64	5.34	4.81	40		
Urban intersection 3	1.53	3.64	4.86	4.81	45		
Urban	0.10	3.03	5.24	7.40	120	500 MHz	Ref. [29]
Suburban	1.47	6.63	6.42	9.22	200		
Urban	1.60	2.20	3.14	4.50	35	725 MHz	Ref. [30]
**Scenario**	**Conditions**	$\gamma_{1}$	$\gamma_{2}$	$\sigma_{S}$ **(dB)**	$d_{C}$ **(m)**	**Frequency Band**	**Related Works**
Highway	LOS	1.66	2.88	3.95	104	5.6 GHz	Ref. [18]
	OLOS	----	3.18	6.12			
Urban	LOS	1.81	2.85	4.15	104		
	OLOS	1.93	2.74	6.67	104		

## Data Availability

The original contributions presented in the study are included in the article, further inquiries can be directed to the corresponding author.
